# Multimorbidity Among Urban Poor in India: Findings From LASI, Wave-1

**DOI:** 10.3389/fpubh.2022.881967

**Published:** 2022-06-02

**Authors:** Abhinav Sinha, Sushmita Kerketta, Shishirendu Ghosal, Srikanta Kanungo, Sanghamitra Pati

**Affiliations:** Department of Public Health, ICMR-Regional Medical Research Centre, Bhubaneswar, India

**Keywords:** multimorbidity, LASI, India, inequity, urban poor, UHC

## Abstract

**Background:**

Multimorbidity has become a norm in low-and middle-income countries such as India requiring notable health system improvements to combat. Urban population is a heterogeneous group where poor are at a risk of facing inequity in accessing healthcare services which can jeopardize our efforts to attain universal health coverage (UHC). We aimed to estimate the prevalence, assess correlates and patterns of multimorbidity among urban poor. Further, we assessed the outcomes of multimorbidity such as healthcare utilization, expenditure and self-rated health.

**Methods:**

Longitudinal Aging Study in India (LASI), wave-1 is a nationally representative survey conducted amongst participants aged ≥45 years in 2017–18. We included 9,327 participants residing in urban areas, categorized as poor based on monthly per capita expenditure. Descriptive statistics computed prevalence with 95% uncertainty interval. Multivariable logistic regression was executed to assess the association between multimorbidity and various correlates, expressed as adjusted odds ratio. An ordinal regression model was run between self-rated health and number of chronic conditions.

**Results:**

The prevalence of multimorbidity was 45.26% among the urban poor. Hypertension and oral morbidities were the most commonly observed dyad. Respondents who were poorer [AOR: 1.27 (1.06–1.51)] had higher chances of having multimorbidity than the poorest. Respondents with a health insurance [AOR: 1.40 (1.14–1.70)] had a higher risk of having multimorbidity. In-patient admission was significantly higher among participants having multimorbidity. Out of pocket expenditure increased while self-rated health deteriorated with each additional morbid condition.

**Conclusion:**

Multimorbidity is found to be increasingly prevalent among urban poor and individuals having health insurance which demonstrates the need to expand healthcare insurance schemes such as Ayushman Bharat for urban poor to achieve UHC.

## Introduction

Multimorbidity, the co-occurrence of two or more chronic conditions being related or not, in an individual is evidently rising among aging population (1, 2). It has emerged as a significant global health issue that necessitates a comprehensive care strategy and notable healthcare system advancements to combat. The burden of multimorbidity is not limited to the high income countries alone, rather is equally observed in the low-and middle-income countries (LMICs) owing to the changing lifestyle and cultural behaviors, urbanization, and changes in environmental exposures (3). Our previous systematic review estimated the prevalence of multimorbidity in South Asia to range from 4.5% to 83% across different age groups (4). Multimorbidity is often associated with an increased healthcare utilization, compromised physical functioning and quality of life, and psychological distress among patients and families (5). Additionally, polypharmacy has been detrimental to patient adherence and health (6).

The increasing burden of multimorbidity has a significant impact on the ability to achieve universal health coverage (UHC) (7). Although, healthcare facilities in urban areas are developing quicker than rural but, due to growing urbanization and migration, urban population has become a heterogeneous group where poor are compelled to face disparities in accessing sanitation, water, housing, and healthcare services (8). Many urban households face a threat of impoverishment due to increased out of pocket expenditure while seeking healthcare and loss of income due to illness (9, 10). They often spend 70% of total out of pocket expenditure on medicine purchases in India (10). Additionally, evidence suggests the onset of multimorbidity tends to occur 10–15 years earlier among people residing in the most deprived areas than those in the most affluent posing a challenge for this vulnerable group (11, 12).

Usually, in the areas of high socio-economic deprivation such as urban poor, the difficulties associated with multimorbid patients are exacerbated by the so-called 'inverse care law,' which states that the rising requirements and demands result in decreased healthcare access, fewer consultations, decreased patient enablement, and increased physician stress (13). As a result of the increasing treatment burden and lower capacity to cope, mental health problems such as depression and anxiety are exacerbated (14). Due to the limited access to healthcare, poor population have a combined burden of infectious and chronic diseases and poor health and quality of life outcomes (15). A study on multimorbidity in extreme poverty estimated the prevalence of two or more chronic illnesses at 22.8 % (16).

The growing burden of multimorbidity may severely impact the achievement of UHC, particularly among older adults and urban poor (17). Inadequately integrated and disease-specific care management programs such as National Programme for Prevention and Control of Cancer, Diabetes, Cardiovascular Diseases and Stroke (NPCDCS) may accentuate these issues, impeding the provision of quality care. Hence, there is a significant unmet need for accessible, egalitarian, and responsive primary healthcare services among vulnerable groups such as urban poor. Recent programmes such as Ayushman Bharat envisage to provide continuum of care for vulnerable sections through health insurance which further needs evidence based guidance to inform decisions about service provision and resource allocation. Hence, we aimed to estimate the prevalence, assess correlates and frequently occurring patterns of multimorbidity among poor residing in urban areas. Further, we aimed to assess the patient outcomes of multimorbidity such as healthcare utilization, expenditure and self-rated health.

## Methods

### Study Design and Participants

We conducted a population-based, cross-sectional analysis using data from the Longitudinal Aging Study in India (LASI), wave 1. LASI is a biennial national survey conducted during April 2017–December 2018, to provide estimates on the indicators of the health, economics, and social behaviors of aging population in India. LASI adopted a multistage stratified area probability cluster sampling design to arrive at a nationally representative sample of 72,250 individuals aged ≥45 years and their spouses (irrespective of age). LASI conducted face to face participant interviews using a pre-validated questionnaire in the local language of the respective states. LASI did not include persons living in collective arrangements such as nursing homes, jails, army camps, care homes etc. Individual response rate to LASI, wave 1 was 87.3%. To ensure standardization, LASI followed comprehensive manuals for field protocol, procedures, quality control and control of non-sampling errors. At national level, training was provided to trainers followed by state level training of investigators, The detailed description of methodology can be referred from LASI wave-1, India report (18).

### Procedures

**Figure 1:**
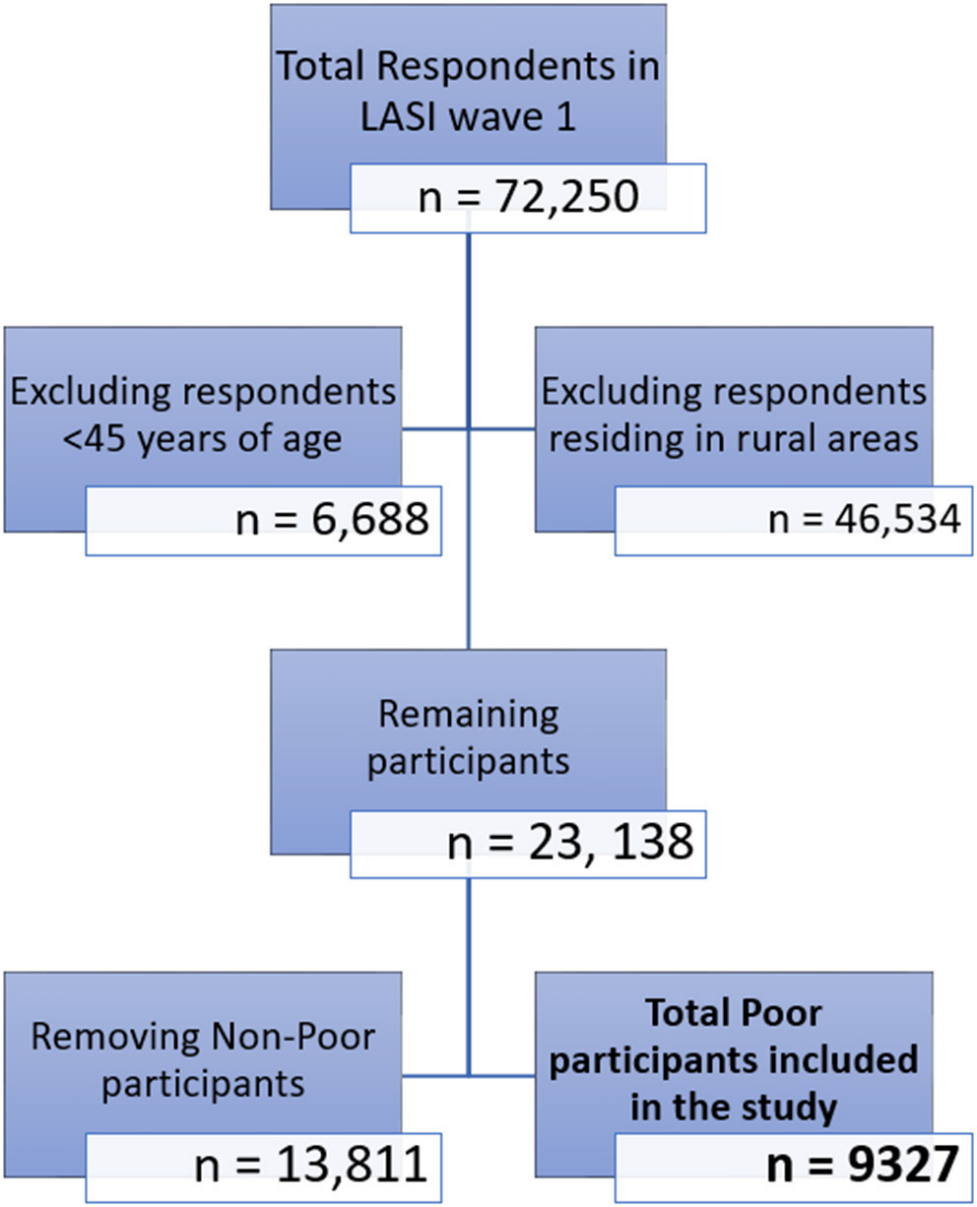
Sample size derivation.

For this study, we included data from 9,327 participants aged ≥45 years clubbed from “poorer” and “poorest” quintiles based on MPCE (monthly per capita expenditure), residing in urban areas termed as urban poor. The sample size of 9,327 (12.91 % of total participants) was reached after excluding a total 62,923 participants who were either below 45 years of age, resided in rural areas or did not belong to the poorer or poorest MPCE quintile. A brief explanation on inclusion criteria for this study is provided in Figure 1.

### Variables

#### Socio-Demographic and Socio-Economic Characteristics

Various socio-demographic characteristics such as sex (male and female), region (East, West, North, South, North-east & Central) and social groups (scheduled tribe, scheduled caste, other backward class and others) were included in the analysis. Age was categorized into three groups viz., 45–59 years, 60–74 years, and ≥75 years. Education was derived from the self-reported highest level of education and was regrouped as “no formal education”, “up to primary”, “middle school to higher secondary and diploma”, and “graduation and above”. Occupation was predicated from past work engagement for at least three consecutive months in the lifetime and current working status; categorized into three groups “never worked”, “currently working”, and “currently not working”. Further, depending on the living arrangements with participant's partner, we grouped them as “with partner” and “without partner”.

Healthcare cost incurred which includes healthcare provider fess, medicines, diagnostic tests, hospitalization charges, transportation, and other expenses due to hospital visit were elicited from respondents in Indian Rupees (₹). Healthcare utilization was assessed through the number of hospitalizations and out-patient visits in the past 12 months from the date of interview. Based on the total number of visits they were classified as “never” or “at least once”. To estimate the healthcare access by the participants LASI, enquired about the type of facility visited and healthcare providers available during their visits. Further, self-rated health was used as a proxy indicator for health-related quality of life (HRQoL) computed on the basis of five-point Likert scale (“very good”, “good”, “fair”, “poor”, and “very poor”) administered for subjective assessment of health status.

#### Outcome

The primary outcome of our study was multimorbidity, defined as the co-occurrence of two or more chronic conditions within an individual, without defining an index disease. We considered 17 self-reported chronic conditions (hypertension, diabetes, cancer, chronic lung disease, chronic heart disease, stroke, arthritis or bone/joint conditions, psychological or neurological conditions, hypercholesterolemia, thyroid disorder, gastrointestinal problems, skin disease, chronic kidney disease, urine incontinence, oral morbidities, visual impairment, and hearing impairment) to attain the outcome variable. Number of morbidities was scored as one in presence of one chronic condition, and sum of these scores was generated for each observation. As defined above, individuals scoring at least two or more were identified as having multimorbidity.

### Statistical Analysis

The analysis was done using STATA statistical software version 17.0 for Windows (Stata Corp, College Station, TX, US). We used descriptive statistics (frequency & percentage) to describe the characteristics of study population. They were further used to describe the healthcare utilization & access. Prevalence of multimorbidity across various attributes was determined along with 95% confidence intervals (CI) as a measure of uncertainty for weighted proportions. We computed crude odds ratio (OR) to assess association between individual characteristics and multimorbidity followed by a multivariable logistic regression model to assess the potential risk factors associated with multimorbidity, reported as adjusted odds ratio (AOR) with 95% CI. Patterns (dyads and triads) of co-occurring conditions were assessed through a simple matrix approach which employed a comprehensive analysis of all possible and exhaustive combinations of chronic conditions with frequency of more than 0.5% (19). Next, median healthcare cost incurred by these patterns were estimated for those who spent on health. Further, an ordinal regression was computed between self-rated health with number of morbid conditions and adjusted for other covariates. Survey weights were considered during analysis to compensate for complex survey designs.

### Ethical Considerations

This study is based on anonymous data obtained from LASI. The original LASI study was approved by the human ethics committee of Indian Council of Medical Research, New Delhi and International Institute of Population Sciences, Mumbai. Informed written consent was obtained from all participants prior to participation.

## Results

### Description of the Study Population

Our study is based on a total of 9,327 participants. The mean age of respondents was 59.6 ± 10.4 years. Women comprised 55.7% of the study population. We observed 40.63% of the participants had no formal education while only 16.30% of the participants had health insurance. The detailed description of sample characteristics is provided in Table 1.

**Table 1:** Weighted socio-demographic characteristics of the study population and prevalence of multimorbidity across various socio-demographic attributes.

**Attributes**	**Categories**	***n* (%)**	**Multimorbidity present *n*, % (95% CI)**
Age group (years)	45–59	4,775 (51.20)	1,616, 33.83 (32.50–35.21)
	60–74	3,545 (38.01)	1,893, 53.41 (51.74–55.05)
	≥75	1,007 (10.79)	646, 64.14 (61.10–67.12)
Gender	Male	4,131 (44.29)	1,696, 41.06 (39.55–42.57)
	Female	5,196 (55.71)	2,458, 47.31 (45.94–48.67)
Caste	Scheduled caste	1,487 (16.13)	640, 43.07 (40.51–45.60)
	Scheduled tribe	370 (4.01)	141, 38.16 (33.14–43.27)
	Other backward class	4,561 (49.49)	1,975, 43.30 (41.86–44.75)
	Others	2,799 (30.37)	1,352, 48.30 (46.44–50.17)
Region	East	1,560 (16.73)	822, 52.68 (50.18–55.19)
	West	2,417 (25.91)	936, 38.73 (36.78–40.70)
	North	675 (7.24)	329, 48.71 (44.91–52.58)
	South	2,516 (26.97)	1,218, 48.42 (46.44–50.38)
	North east	186 (2.00)	74, 39.53 (32.70–47.20)
	Central	1,973 (21.15)	776, 39.34 (37.17–41.53)
Education	No formal education	3,789 (40.63)	1,720, 45.39 (43.80–47.00)
	Primary completed	2,585 (27.71)	1,252, 48.43 (46.49–50.38)
	Up to matriculation	1,987 (21.31)	831, 41.85 (39.64–44.03)
	Higher secondary and diploma	464 (4.98)	170, 36.54 (32.24–41.20)
	Graduate and above	501 (5.37)	181, 36.16 (31.91–40.51)
Working status	Never worked	2,943 (31.57)	1,584, 53.84 (52.00–55.64)
	Currently working	3,624 (38.87)	1,141, 31.48 (29.97–33.02)
	Currently not working	2,755 (29.56)	1,428, 51.84 (49.95–53.71)
Partner	With partner	6,614 (70.92)	2,795, 42.26 (41.07–43.47)
	Without partner	2,712 (29.08)	1,359, 50.12 (48.21–52.01)
MPCE quintiles	Poorer	4,831 (51.79)	2,002, 41.44 (40.05–42.85)
	Poorest	4,496 (48.21)	2,153, 47.88 (46.42–49.36)
Health insurance	Yes	1,498 (16.30)	760, 50.70 (48.17–53.30)
	No	7,691 (83.70)	3,333, 43.34 (42.23–44.45)

### Profile and Distribution of Morbidity

The overall prevalence of multimorbidity in the study population was estimated to be 45.26%. Multimorbidity was more common among participants aged ≥75 years, females, who lived without partner and had a health insurance. Multimorbidity was found to be more prevalent among the non-working group such as 53.84% amongst participants who never worked and 51.84% among participants who are currently not working. Southern region accounted for the highest prevalence of multimorbidity while more than half of the Eastern region had multimorbidity. Amongst the urban poor, poorest had the higher prevalence of multimorbidity (47.88%) as compared to the poorer (41.44%). The detailed description of prevalence of multimorbidity across various socio-demographic variables is presented in Table 1.

We observed hypertension and oral morbidities s (3.4%) were the most commonly occurring dyads followed by hypertension and diabetes (2.07%) while hypertension, diabetes and oral morbidities (1.04%) formed the most commonly presenting triad (Table 2).

**Table 2:** Frequently occurring multimorbid patterns (frequency > 0.5%).

**Sl. No**	**Patterns**	**Frequency**	**Percentage**
1	Hypertension + Oral Conditions	321	3.44
2	Hypertension + Diabetes	193	2.07
3	Gastrointestinal disease + Oral Conditions	179	1.92
4	Arthritis and other bone/joint diseases + Oral Conditions	154	1.65
5	Hypertension + Diabetes + Oral Conditions	97	1.04
6	Hypertension + arthritis and other bone/joint diseases	89	0.96
7	Hypertension + Gastrointestinal disease	86	0.92
8	Hypertension + gastrointestinal disease + Oral Conditions	77	0.82
9	Diabetes + Oral Conditions	75	0.81
10	Hypertension + Arthritis and other bone/joint diseases + Oral Conditions	73	0.78
11	Arthritis and other bone/joint diseases + Gastrointestinal disease	71	0.76
12	Hypertension + Visual impairment	57	0.61
13	Chronic Lung disease + Oral Conditions	49	0.53
14	Hearing impairment + Oral Conditions	48	0.51
15	Hypertension + Skin diseases	47	0.5

### Correlates of Multimorbidity

The crude odds ratio showed a significant association between age, gender, primary education and no work with multimorbidity. The findings from multivariable analysis suggested the likelihood of having multimorbidity increased with aging as the participants aged 60–75 years had [AOR: 2.01 (1.67–2.43)] which increased to [AOR: 2.76 (2.02–3.75)] among respondents aged ≥75 years. Respondents from eastern part of the country had the highest chances of having multimorbidity [AOR: 2.05 (1.44–2.92)] followed by south [AOR: 1.74 (1.20–2.54)]. We observed the risk of multimorbidity to be highest among participants who had never worked in their lifetime for at least three consecutive months with an AOR of 2.16 (1.70–2.76). Respondents who had health insurance coverage had a higher risk of having multimorbidity [AOR; 1.40 (1.14–1.70)] as compared to the others. The univariable analysis revealed a protective association for multimorbidity but after adjusting for other covariates, multivariable regression depicted multimorbidity to be significantly common [AOR: 1.27 (1.06–1.51)] amongst poorer as compared to the poorest group (Table 3).

**Table 3:** Logistic regression showing association between multimorbidity and its correlates.

**Attributes**	**Categories**	**OR (95% CI)**	***p*-values**	**AOR (95% CI)**	***p*-value**
Age groups (years)	45– 60	Reference		Reference	
	60–75	2.24 (1.87–2.68)	0.000	2.01 (1.67–2.43)	0.000
	≥75	3.50 (2.63–4.64)	0.000	2.76 (2.02–3.75)	0.000
Gender	Male	Reference		Reference	
	Female	1.29 (1.09–1.53)	0.004	0.94 (0.73–1.20)	0.598
Caste	Scheduled caste	0.99 (0.78–1.26)	0.939	1.05 (0.84–1.31)	0.681
	Scheduled tribe	0.81 (0.58–1.13)	0.208	1.05 (0.73–1.50)	0.805
	Other backward class	Reference		Reference	
	Others	1.22 (1.01–1.48)	0.040	1.17 (0.97–1.41)	0.096
Region	East	1.70 (1.23–2.36)	0.001	2.05 (1.44–2.92)	0.000
	West	0.97 (0.70–1.34)	0.839	1.05 (0.74–1.49)	0.777
	North	1.45 (1.05–2.00)	0.023	1.72 (1.20–2.47)	0.003
	South	1.44 (0.98–2.09)	0.059	1.74 (1.20–2.54)	0.004
	North east	Reference		Reference	
	Central	0.99 (0.72–1.36)	0.962	1.14 (0.81–1.62)	0.449
Education	No formal education	1.47 (0.95–2.28)	0.087	1.17 (0.75–1.84)	0.492
	Primary completed	1.66 (1.07–2.58)	0.025	1.37 (0.88–2.14)	0.169
	Up to matriculation	1.27 (0.81–2.00)	0.300	1.15 (0.74–1.78)	0.536
	Higher secondary and diploma	1.02 (0.58–1.78)	0.954	1.01 (0.59–1.71)	0.984
	Graduate and above	Reference		Reference	
Working status	Never worked	2.54 (2.08–3.10)	0.000	2.16 (1.70–2.76)	0.000
	Currently working	Reference		Reference	
	Currently not working	2.34 (1.88–2.91)	0.000	1.75 (1.38–2.23)	0.000
Partner	With partner	Reference		Reference	
	Without partner	1.37 (1.13–1.67)	0.002	0.92 (0.75–1.12)	0.393
Healthcare insurance	Yes	1.34 (1.13–1.61)	0.001	1.40 (1.14–1.70)	0.001
	No	Reference		Reference	
MPCE quintile	Poorer	0.77 (0.65–0.91)	0.003	1.27 (1.06–1.51)	0.010
	Poorest	Reference		Reference	

### Patient Outcomes of Multimorbidity

#### Trends in Healthcare Utilization and Expenditure

We observed a significantly higher in-patient (IPD) admission among those with multimorbidity (10.94% vs. 5.81%) as compared to those without multimorbidity while visit to the out-patient department (OPD) was almost similar. Participants with (53.69%) or without multimorbidity (37.53%) preferred private hospitals over public facilities. Majority of the participants uniformly had access to the doctors. We found less than one fifth of the study population were insured under any healthcare insurance scheme which was mostly funded by state government (Table 4).

**Table 4:** Trends of health care utilization among urban poor.

**Healthcare access**	**Multimorbidity present**		
	**Absent *n*, % (95% CI)**	**Present *n*, % (95% CI)**	***p*-value**
**OPD visits in last 12 months (*****n** **=*** **5,941)**			
Never	579 20.77 (19.28–22.32)	579 18.35 (17.02–19.75)	0.000
At least once	2,208 79.23 (77.67–80.72)	2,575 81.65 (80.25–82.98)	
**IPD visits in last 12 months (*****n** **=*** **6,034)**			
Never	2,667 94.19(93.25–95.00)	2,852 89.06 (87.94–90.13)	0.000
At least once	165 5.81 (4.99–6.75)	350 10.94 (9.87–12.06)	
**Type of health care facility (*****n** **=*** **9,182)**			
Public	689 13.53 (12.60–14.50)	742 18.14 (16.98–19.36)	0.000
Private	1,911 37.53 (36.19–38.87)	2,196 53.69 (52.14–55.22)	
Others	416 8.17 (7.43–8.95)	495 12.11 (11.12–13.14)	
None	2,076 40.77 (39.41–42.13)	657 16.06 (14.95–17.23)	
**Healthcare provider (*****n** **=*** **9,190)**			
Doctors	2,340 45.93 (17.74–20.16)	2,797 68.31 (66.85–69.73)	0.000
Nurses	9 0.17 (0.08–0.34)	11 0.27 (0.13–0.48)	
Paramedics	449 8.81 (8.05–9.62)	393 9.60 (8.71–10.54)	
Traditional	70 1.37 (1.07–1.73)	77 1.88 (1.49–2.34)	
Others	57 1.13 (0.84–1.45)	42 1.02 (0.74–1.38)	
None	2,170 42.60 (41.23–43.96)	775 18.92 (17.74–20.16)	
**Health insurance (*****n** **=*** **9,189)**			
Yes	738 14.49 (13.53–15.48)	760 18.56 (17.39–19.79)	0.022
No	4,358 85.51 (84.52–86.65)	3,333 81.44 (80.21–82.61)	
**Source of health insurance (*****n** **=*** **1,695)**			
Central govt.	225 27.08 (24.11–30.27)	257 29.99 (26.94–33.18)	0.700
State govt.	494 59.5 (56.09–62.88)	503 58.76 (55.31–62.01)	
Private	60 7.27 (5.56–9.21)	64 7.48 (5.80–9.44)	
Others	51 6.10 (4.61–8.00)	32 3.77 (2.57–5.23)	

**Figure 2:**
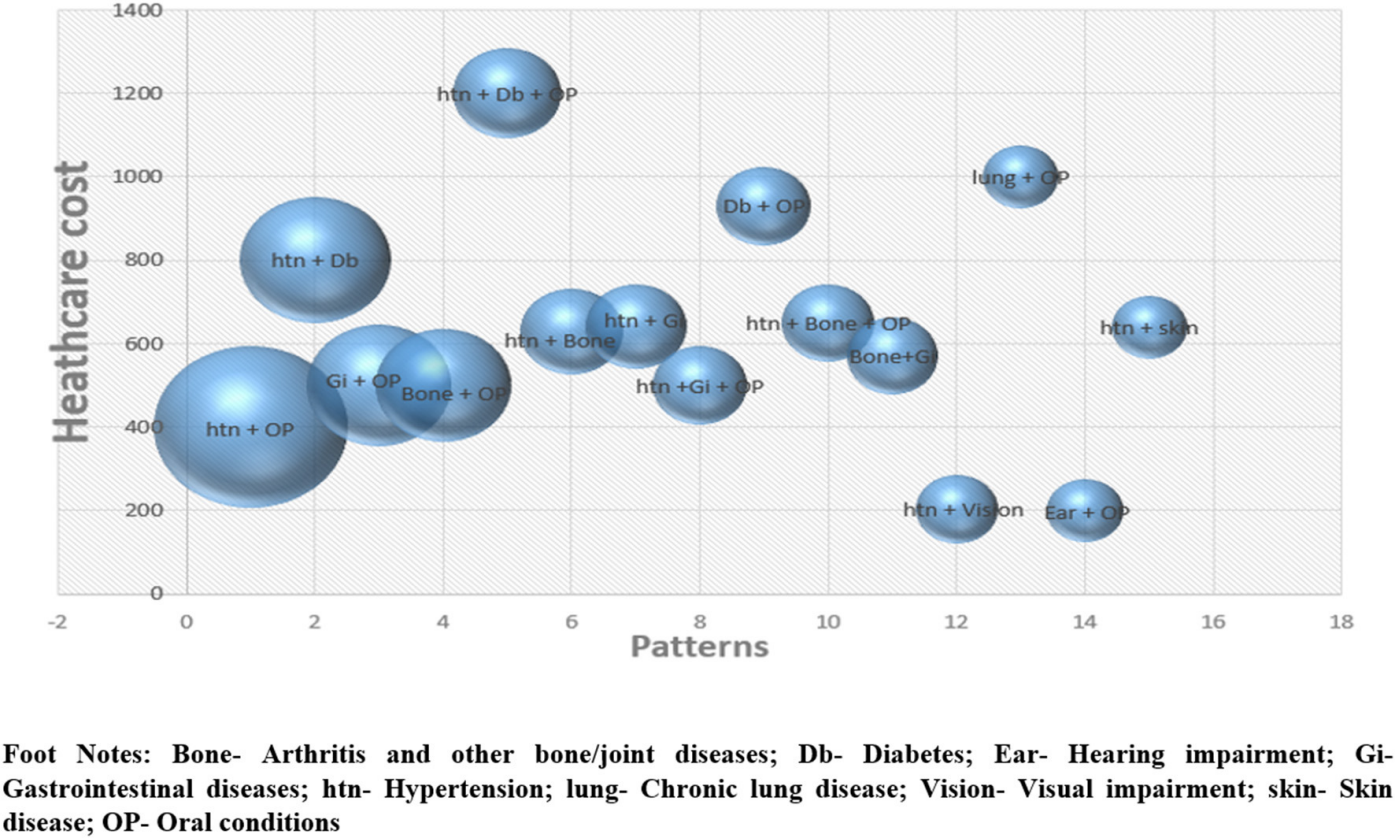
Healthcare expenditure across commonly occurring patterns of multimorbidity.

**Figure 3:**
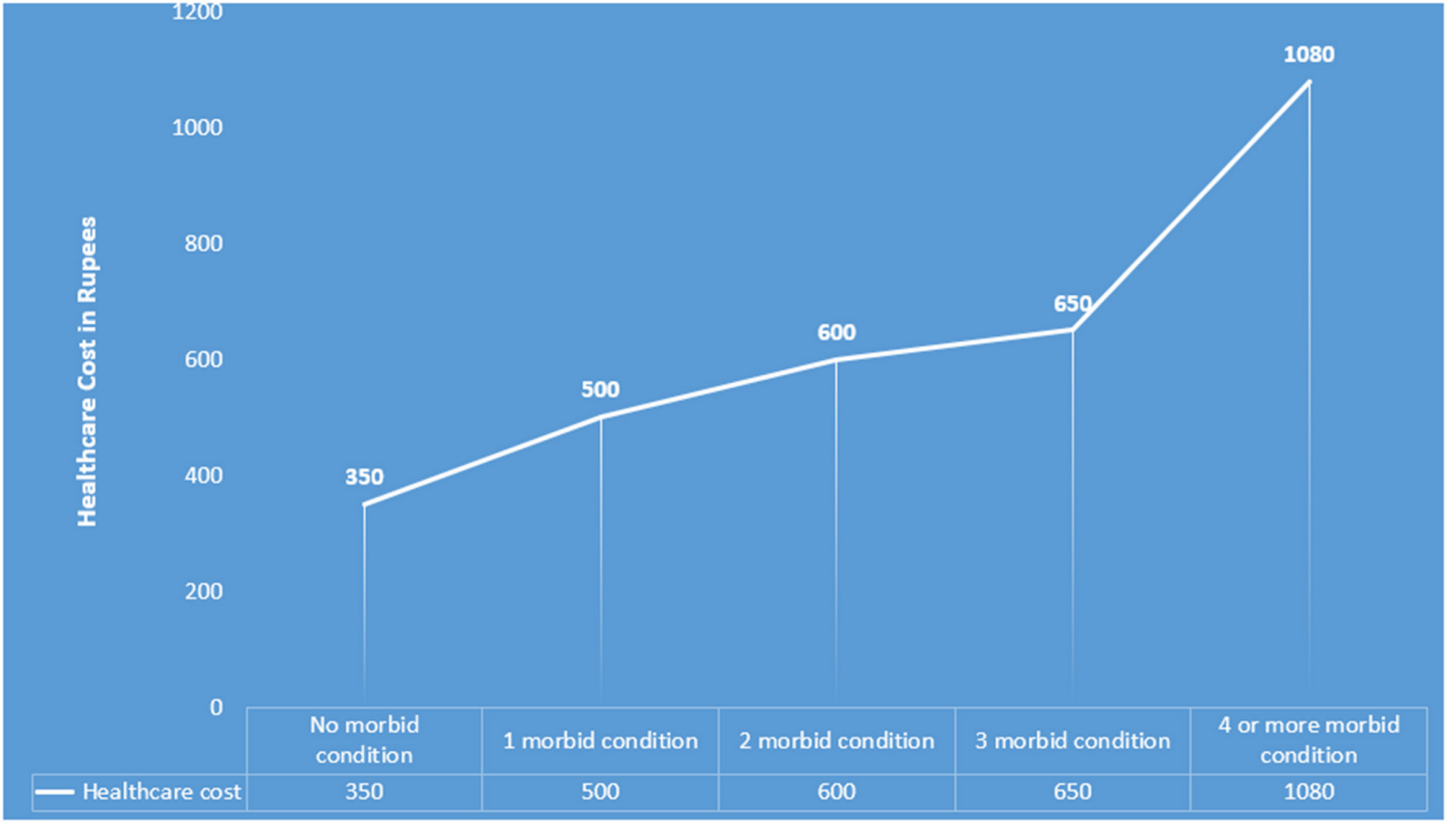
Trends of healthcare expenditure with number of morbid conditions among urban poor (median INR).

We plotted the patterns of commonly occurring chronic conditions with the healthcare cost incurred for them. It depicted that the participants spent a maximum of around INR 1,200 for the triad of hypertension, diabetes and oral morbidities followed by INR 1,000 for dyad of chronic lung condition and oral morbidities (Figure 2). We observed an increase in out of pocket expenditure (OOPE) for each additional long term condition. The total median healthcare expenses (in INR) among participants with no morbid condition was 350 which increased to 600 and 1,080 for two and four or more chronic conditions respectively (Figure 3).

#### Self-Rated Health

Our findings from ordinal regression model suggested, self-rated health deteriorated with an increase in each additional chronic condition (Table 5). After adjusting for various attributes such as age, gender, and health insurance etc.; we found a significant per unit deterioration of self-rated health as the number of morbid conditions increased with highest among those with four or more morbid conditions [AOR:9.75 (7.34–12.96)].

**Table 5:** Ordered logistic regression showing association between self-rated health with number of morbid conditions.

**Attributes**	**Categories**	**AOR, 95% CI**	***p*-value**
Number of morbid	No morbid conditions	Reference	
conditions	One morbid condition	1.68 (1.33–2.11)	0.000
	Two morbid conditions	2.66 (2.10–3.37)	0.000
	Three morbid conditions	4.92 (3.63–6.66)	0.000
	Four or more morbid conditions	9.75 (7.34–12.96)	0.000

^*^Adjusted for age, gender, caste, region, education, working status, partner, MPCE quintiles, and health insurance.

## Discussion

Universal health coverage aims to provide accessible and affordable quality health services for every person everywhere (20). The Sustainable Development Goal 3 calls on to prioritize health for all by 2,030 where vulnerable populations such as poor need special attention (21). Estimates suggest, around 90% of the world's poorest billion live in sub-Saharan Africa or South Asia (22). In India, a South Asian country urban population is projected to rise from 31% in 2011 to 46% by 2030 out of which 30%–40% people reside in slums or areas of deprivation (23). Additionally, the healthcare system is primarily oriented toward management of infectious diseases along with maternal and child health (22) whereas multimorbidity requires long term support where continuity of care plays an important role (24). We estimated the magnitude and assessed patient outcomes of multimorbidity among urban poor to orient evidence based resources for this group.

Our study elicited a high prevalence of multimorbidity among poor population residing in urban areas. Multivariable regression revealed a higher chance of having multimorbidity among poorer group than the poorest; and participants having healthcare insurance. Healthcare expenditure increased with each additional condition and was highest for the most commonly occurring triad. A significantly higher healthcare utilization such as IPD visit was observed among participants having multimorbidity. Self-rated health deteriorated with each additional chronic condition.

We observed an overall prevalence of multimorbidity to be around 45% which is consistent with the findings of our previous community based study which reported 41% prevalence among participants aged ≥18 years in urban areas of Odisha, India (25). We obtained a slight higher prevalence in the present study which may be due to the aging population considered here. Moreover, a study from China reported that the impact of multimorbidity on work productivity loss was found lesser among rural residents than the urban group (26). Additionally, a study conducted among urban residents of Spain found almost 50% of the residents had multimorbidity (27). These findings are almost similar to our findings however, there is a paucity of literature on multimorbidity among urban poor in LMICs such as India which makes it challenging to compare our results with similar studies (28).

After adjusting for other variables, we found a higher risk of multimorbidity among poorer group than the poorest. This points toward the heterogeneity within urban poor in accessing healthcare facilities. A probable explanation for this could be that the urban poor are mostly migrants coming for employment opportunities to big cities where neo-migrants often lack access to healthcare facilities or are often not covered in the local list of frontline workers such as ASHA which devoid them from obtaining primary care facilities (29, 30). Additionally, a systematic review suggested higher odds of multimorbidity among urban residents (31). Nonetheless, lack of healthcare insurance is another factor for under diagnosis of multimorbidity among the poor group as elucidated by our study. We observed multimorbidity to be more common amongst respondents who had a healthcare insurance which clearly elucidates better prospects of receiving healthcare facilities among people with insurance. This is similar to the findings of our previous study which also revealed participants without insurance had a negative association with multimorbidity (32). Here, it is worth noting that implementing equitable and egalitarian health insurance schemes such as Ayushman Bharat needs special attention for inclusion of this group.

Furthermore, we found an increase in healthcare expenditure with each additional condition which is consistent with the findings of a previous study which showed mean out of pocket expenditure for OPD visit increased from INR 262.2 for participants with no NCD to INR 431 amongst those who had multimorbidity. Here, it is worth noting that higher OOPE implies a need to pay directly from pocket for utilizing healthcare facilities which gets hampered by the inability to pay as in the case of urban poor (33). Multimorbidity requires long term use of multiple drugs, periodic investigations and visits to doctor (34) which is easy for poorer group, an explanation for easy access to healthcare and hence, better diagnosis and continuity of care among this group than the poorest. Additionally, older adults are often dependent on their children for finances which compels them to underutilize healthcare services (35).

Previous studies document an increased hospital admission and emergency visits among patients with multimorbidity (32). We also observed a significantly higher IPD visit among multimorbid individuals which further needs attention for multimorbidity management especially amongst urban poor who face disparity in healthcare utilization and expenditure. With the change in disease epidemiology, it is imperative to prioritize quality health services through infrastructure development and capacity building of human resources with an aim to reduce OOPE. Additionally, reinforcing trust in public health system is necessary to align with the global discourse of attaining UHC. Furthermore, self-rated health (SRH), a proxy indicator for quality of life was found to deteriorate with each additional chronic condition which is in harmony with the findings of previous studies which revealed patients with multimorbidity have inferior self-rated health (36).

### Implications for Policy and Practice

Multimorbidity is found to be increasingly prevalent among urban poor and those who are not working which points toward an unmet need for multimorbidity care among this marginalized group. The existing Ayushman Bharat scheme envisage to provide free healthcare services to poor, but its frame needs to be broadened to cover urban poor specially the migrating population. It is imperative to have Health and Wellness Centers (HWCs) in the vicinity of urban slums so as to have easy access to health services thus reducing OOPE. A focus on older adults and people living in eastern region is necessary. Additionally, risk factors of multimorbidity such as dietary control, abstaining from tobacco, alcohol and physical activities should be targeted at an early state to prevent or delay the disease onset. Screening should be expanded in current programs such as the National Programme for Prevention and Control of Cancer, Diabetes, Cardiovascular Diseases, and Stroke (NPCDCS). There is an urgent need to create awareness regarding oral hygiene from childhood. Information, education and communication activities should aim at motivating people to equally prioritize oral health in order to improve oral hygiene. Also, coordinated quality care for both physical and oral health should preferably be provided under one umbrella. A holistic care approach for women's overall well-being is required as women have been identified to be at higher risk of having multimorbidity. Integration of current programs such as NPCDCS and RMNCH+A will be useful for continued support to women's health. Digital literacy to avail e-services is required especially among women and older adults as these groups may face challenges in accessing healthcare due to inadequate digital literacy. Further, cohort studies are warranted to understand the heterogeneity in healthcare needs within urban poor.

### Strengths and Limitations

This study used a national level data to extrapolate the differences in multimorbidity prevalence and its outcomes among urban poor, a vulnerable group which needs special attention for healthcare to achieve UHC. We used 16 chronic conditions to assess multimorbidity which is another strength of this study, but these conditions were self-reported which might undermine the true population estimates. Evidence suggests self-reported conditions leads to misclassification bias which under-represents the issue being studied (37, 38). However, previous studies have also suggested a high agreement between self-reported conditions and clinical assessed conditions (39). Nonetheless, self-reported conditions majorly limit our study. Furthermore, this study was based on cross-sectional data which could not establish causality.

## Conclusion

Our findings elicit inequalities in the magnitude and distribution of multimorbidity within urban poor. Participants having health insurance had higher chances of having multimorbidity which can be due to better healthcare access in this group. Additionally, poorer SRH and higher OOPE with each additional long term condition cannot be overlooked. This points toward expanding coverage of healthcare insurance schemes such as Ayushman Bharat along with setting HWCs for urban poor to achieve UHC.

## Declaration

All methods were carried out in accordance with relevant guidelines and regulations.

## Data Availability Statement

Publicly available datasets were analyzed in this study. This data can be found here: https://iipsindia.ac.in/content/lasi-wave-i.

## Ethics Statement

As the present study utilizes de-identified data from a secondary source available in public domain, it doesn't require ethical review and approval. Written informed consent for participation was not required for this study in accordance with the national legislation and the institutional requirements.

## Author Contributions

SP: concept and design. AS, SKe, and SG: acquisition, statistical analysis, or interpretation of data. AS, SKe, and SG: drafting of the manuscript. SP and SKa: monitored analysis and critical revision of the manuscript for important intellectual content. SP and SKa: administrative and technical support. SP: supervision. All authors reviewed the manuscript. All authors contributed to the article and approved the submitted version.

## Conflict of Interest

The authors declare that the research was conducted in the absence of any commercial or financial relationships that could be construed as a potential conflict of interest.

## Publisher's Note

All claims expressed in this article are solely those of the authors and do not necessarily represent those of their affiliated organizations, or those of the publisher, the editors and the reviewers. Any product that may be evaluated in this article, or claim that may be made by its manufacturer, is not guaranteed or endorsed by the publisher.

## Abbreviations

LMICs: Low-and-middle income countries
UHC: Universal Health Coverage
HWCs: Health and Wellness Centers
NCDs: Non-communicable diseases
SRH: Self-rated health
OOPE: Out of pocket expenditure
NPCDCS: National Programme for Prevention and Control of Cancer, Diabetes, Cardiovascular Diseases and Stroke
LASI: Longitudinal Aging Study in India
MPCE: Monthly per capita income
OPD: Out-patient department
IPD: In-patient department
HRQoL: Health related quality of life
CI: Confidence Interval
OR: Odds ratio
AOR: Adjusted Odds Ratio
INR: Indian National Rupees.

## References

[1] MacMahon S Calverley P Chaturvedi N Chen Z Corner L Davies M. Multimorbidity: A Priority for Global Health Research. London: The Academy of Medical Sciences (2018). p. 127.

[2] Pati S Swain S Hussain MA Kadam S Salisbury C. Prevalence, correlates, and outcomes of multimorbidity among patients attending primary care in Odisha, India. Ann Fam Med. (2015) 13:446–50. 10.1370/afm.1843 26371265 PMC4569452

[3] Oni T Unwin N. Why the communicable/non-communicable disease dichotomy is problematic for public health control strategies: implications of multimorbidity for health systems in an era of health transition. Int Health. (2015) 7:390–9. 10.1093/inthealth/ihv040 26103981 PMC4638105

[4] Pati S Swain S Hussain MA Van Den Akker M Metsemakers J Knottnerus JA. Prevalence and outcomes of multimorbidity in South Asia: a systematic review. BMJ Open. (2015) 5:e007235. 10.1136/bmjopen-2014-007235 26446164 PMC4606435

[5] Marengoni A Angleman S Melis R Mangialasche F Karp A Garmen A. Aging with multimorbidity: a systematic review of the literature. Ageing Res Rev. (2011) 10:430–9. 10.1016/j.arr.2011.03.003 21402176

[6] Davies LE Spiers G Kingston A Todd A Adamson J Hanratty B. Adverse outcomes of polypharmacy in older people: systematic review of reviews. J Am Med Dir Assoc. (2020) 21:181–7. 10.1016/j.jamda.2019.10.022 31926797

[7] Aikins A.D.G. Kushitor M. Koram K. Gyamfi S. Ogedegbe G. Chronic non-communicable diseases and the challenge of universal health coverage: insights from community-based cardiovascular disease research in urban poor communities in Accra, Ghana. BMC Public Health. (2014) 14:1–9. 10.1186/1471-2458-14-S2-S3 25082497 PMC4120153

[8] Agarwal S. The state of urban health in India; comparing the poorest quartile to the rest of the urban population in selected states and cities. Environ Urban. (2011) 23:13–28. 10.1177/0956247811398589

[9] McIntyre D Thiede M Dahlgren G. Whitehead M. What are the economic consequences for households of illness and of paying for health care in low-and middle-income country contexts? Soc Sci Med. (2006) 62:858–65. 10.1016/j.socscimed.2005.07.001 16099574

[10] Garg CC Karan AK. Reducing out-of-pocket expenditures to reduce poverty: a disaggregated analysis at rural-urban and state level in India. Health Policy Plan. (2009) 24:116–28. 10.1093/heapol/czn046 19095685

[11] Barnett K Mercer S Norbury M Watt G Wyke S Guthrie B. The epidemiology of multimorbidity in a large cross-sectional dataset: implications for health care, research and medical education. Lancet. (2012) 380:37–43. 10.1016/S0140-6736(12)60240-2 22579043

[12] Salisbury C Johnson L Purdy S Valderas JM Montgomery AA. Epidemiology and impact of multimorbidity in primary care: a retrospective cohort study. Br J Gen Pract. (2011) 61:e12–21. 10.3399/bjgp11X548929 21401985 PMC3020068

[13] Mercer SW Guthrie B Furler J Watt GC Hart JT. Multimorbidity and the inverse care law in primary care. BMJ. (2012) 344:e4152. 10.1136/bmj.e4152 22718915

[14] Mercer SW Watt GC. The inverse care law: clinical primary care encounters in deprived and affluent areas of Scotland. Ann Fam Med. (2007) 5:503–10. 10.1370/afm.778 18025487 PMC2094031

[15] Agarwal S Satyavada A Kaushik S Kumar R. Urbanization, urban poverty and health of the urban poor: status, challenges and the way forward. Demogr India. (2007) 36: 121–34.

[16] Odland ML Payne C Witham MD Siedner MJ Bärnighausen T Bountogo M. Epidemiology of multimorbidity in conditions of extreme poverty: a population-based study of older adults in rural Burkina Faso. BMJ Glob Health. (2020) 5:e002096. 10.1136/bmjgh-2019-002096 32337079 PMC7170422

[17] Banerjee S. Multimorbidity—older adults need health care that can count past one. Lancet. (2015) 385:587–9. 10.1016/S0140-6736(14)61596-8 25468155

[18] International Institute for Population Sciences (IIPS). Longitudinal Ageing Study in India (LASI). Available online: https://www.iipsindia.ac.in/lasi (accessed November 27, 2021).

[19] Pati S Swain S Metsemakers J Knottnerus JA van den Akker M. Pattern and severity of multimorbidity among patients attending primary care settings in Odisha, India. PLoS ONE. (2017) 12:e0183966. 10.1371/journal.pone.0183966 28910309 PMC5598947

[20] Frenk J De Ferranti D. Universal health coverage: good health, good economics. Lancet. (2012) 380:862–4. 10.1016/S0140-6736(12)61341-5 22959372

[21] Yamey G Shretta R Binka FN. The 2030 sustainable development goal for health. BMJ. (2014) 349:g5295. 10.1136/bmj.g5295 25161288

[22] Bukhman G Mocumbi AO Atun R Becker AE Bhutta Z Binagwaho A. The Lancet NCDI Poverty Commission: bridging a gap in universal health coverage for the poorest billion. Lancet. (2020) 396:991–1044. 10.1016/S0140-6736(20)31907-3 32941823 PMC7489932

[23] Kumar S Kumar S Gupta B. Urban health: needs urgent attention. Indian J Public Health. (2018) 62:214. 10.4103/ijph.IJPH_90_18 30232971

[24] Roland M Paddison C. Better management of patients with multimorbidity. BMJ. (2013) 346:f2510. 10.1136/bmj.f2510 23641032

[25] Pati S Mahapatra P Kanungo S Uddin A Sahoo KC. Managing multimorbidity (multiple chronic diseases) amid COVID-19 pandemic: a community based study from Odisha, India. Front Public Health. (2021) 8:1026. 10.3389/fpubh.2020.584408 33598442 PMC7882709

[26] Zhao Y He L Han C Oldenburg B Sum G Haregu TN. Urban-rural differences in the impacts of multiple chronic disease on functional limitations and work productivity among Chinese adults. Glob Health Action. (2021) 14:1975921. 10.1080/16549716.2021.1975921 34530701 PMC8451617

[27] Violán C Foguet-Boreu Q Roso-Llorach A Rodriguez-Blanco T Pons-Vigués M Pujol-Ribera E. Burden of multimorbidity, socioeconomic status and use of health services across stages of life in urban areas: a cross-sectional study. BMC Public Health. (2014) 14:1–3. 10.1186/1471-2458-14-530 24885174 PMC4060853

[28] Sinha A Varanasi R Pati S. Kaleidoscopic use of World Health Organization's Study on global AGEing and adult health data set to explore multimorbidity and its outcomes in low and middle-income countries: an insider view. J Fam Med Prim Care. (2021) 10:4623–5. 10.4103/jfmpc.jfmpc_1598_21 35280606 PMC8884332

[29] Banerjee A Bhawalkar JS Jadhav SL Rathod H Khedkar DT. Access to health services among slum dwellers in an industrial township and surrounding rural areas: a rapid epidemiological assessment. J Fam Med Prim Care. (2012) 1:20. 10.4103/2249-4863.94444 24478995 PMC3893946

[30] Kusuma YS Kaushal S Garg R Babu BV. Birth preparedness and determinants of birth place among migrants living in slums and slum-like pockets in Delhi, India. Sex Reprod Healthc. (2018) 16:160–6. 10.1016/j.srhc.2018.04.004 29804761

[31] Asogwa OA Boateng D Marzà-Florensa A Peters S Levitt N van Olmen J. Multimorbidity of non-communicable diseases in low-income and middle-income countries: a systematic review and meta-analysis. BMJ Open. (2022) 12:e049133. 10.1136/bmjopen-2021-049133 35063955 PMC8785179

[32] Pati S Agrawal S Swain S Lee JT Vellakkal S Hussain MA. Non communicable disease multimorbidity and associated health care utilization and expenditures in India: cross-sectional study. BMC Health Serv Res. (2014) 14:1–9. 10.1186/1472-6963-14-451 25274447 PMC4283077

[33] Obembe TA Levin J Fonn S. Prevalence and factors associated with catastrophic health expenditure among slum and non-slum dwellers undergoing emergency surgery in a metropolitan area of South Western Nigeria. PLoS ONE. (2021) 16:e0255354. 10.1371/journal.pone.0255354 34464387 PMC8407567

[34] Pati S Mahapatra P Dwivedi R Athe R Sahoo KC Samal M Das RC Hussain MA. Multimorbidity and its outcomes among patients attending psychiatric care settings: an observational study from Odisha, India. Front Public Health. (2020) 8:616480. 10.3389/fpubh.2020.616480 33968863 PMC8096979

[35] Maharana B. Ladusingh L. How does the change in household age-sex composition affect out of pocket healthcare expenditure of older adults in India? Age Int. (2021) 16:1–22. 10.1007/s12126-021-09457-3

[36] Kanungo S Ghosal S Kerketta S Sinha A Mercer SW Lee JT. Association of oral health with multimorbidity among older adults: findings from the longitudinal ageing study in India, Wave-1, 2017–2019. Int J Environ Res Public Health. (2021) 18:12853. 10.3390/ijerph182312853 34886581 PMC8657905

[37] Puri P Singh SK Pati S. Temporal dynamics, patterns and correlates of single and multimorbidity in India, 1994–2018. J Comor. (2021) 11:26335565211062756. 10.1177/26335565211062756 35004339 PMC8728765

[38] Puri P Sinha A Mahapatra P Pati S. Multimorbidity among midlife women in India: well-being beyond reproductive age. BMC Womens Health. (2022) 22:1–5. 10.1186/s12905-022-01693-2 35413903 PMC9004080

[39] Carvalho JN Roncalli ÂG Cancela MD Souza DL. Prevalence of multimorbidity in the Brazilian adult population according to socioeconomic and demographic characteristics. PLoS ONE. (2017) 12:e0174322. 10.1371/journal.pone.0174322 28384178 PMC5383049

